# *Hepatozoon* spp. in stray cats from the metropolitan area of Rio de Janeiro, Brazil[Fn FN1]

**DOI:** 10.1051/parasite/2024026

**Published:** 2024-05-17

**Authors:** Donato Traversa, Angela Di Cesare, Simone Morelli, Barbara Paoletti, Marika Grillini, Antonio Frangipane di Regalbono, Aline da Silva de Mattos Queiroz, Frederic Beugnet, Leonardo Brustenga, Piermarino Milillo, Luciano Antunes Barros

**Affiliations:** 1 Department of Veterinary Medicine, University of Teramo 64100 Teramo Italy; 2 Department of Animal Medicine, Production and Health, University of Padova 35020 Padova Italy; 3 Veterinary University Hospital Universitário Professor Firmino Mársico Filho, Fluminense Federal University Niterói RJ 24230-321 Brazil; 4 Boehringer Ingelheim Animal Health 29 Avenue Tony Garnier 69007 Lyon France; 5 Department of Veterinary Medicine, University of Perugia 06126 Perugia Italy; 6 Azienda Sanitaria Locale (A.S.L.) 75100 Matera Italy; 7 Department of Veterinary Collective Health and Public Health, Fluminense Federal University Niterói RJ 24220-000 Brazil

**Keywords:** Hepatozoonosis, Cats, Tick-borne diseases, Brazil, Genotype, PCR

## Abstract

In the last few years, the number of studies on feline hepatozoonosis has increased, but our knowledge on the actual species of *Hepatozoon* and/or different genotypes affecting felines is still incipient. At least three species, namely *Hepatozoon felis*, *H. canis*, and *H. silvestris*, have been isolated from domestic cats in various countries. Additionally, there are indications that other species and genotypes may affect felines in given geographic areas. This study was carried out to investigate the occurrence of *Hepatozoon* spp. in cats from Niterói, a municipality within the metropolitan area of Rio de Janeiro, Brazil. Individual blood samples were collected from 28 cats enrolled in a spaying/castration program. DNA was extracted from all samples and subjected to sequencing specific for *Hepatozoon* spp. DNA of *H. felis* was found in 21/28 cats (75%), and four genetic polymorphisms never described thus far were detected. This is the first report of *H. felis* in cats living in the State of Rio de Janeiro, and the present data confirm that *H. felis* is a species complex encompassing different genotypes circulating within cat populations. Further studies are warranted to investigate whether different genotypes have different biology or pathogenicity for felids.

## Abstract (fr)

Au cours des dernières années, le nombre d’études sur l’hépatozoonose féline a augmenté, mais nos connaissances sur les espèces d’*Hepatozoon* et/ou différents génotypes affectant les félins sont encore naissantes. Au moins trois espèces, à savoir *Hepatozoon felis*, *H. canis* et *H. silvestris*, ont été isolées chez des chats domestiques dans divers pays. De plus, il semble que d’autres espèces et génotypes puissent affecter les félins dans des zones géographiques données. Cette étude a été réalisée pour étudier la présence d’*Hepatozoon* spp. chez des chats de Niterói, une municipalité de la zone métropolitaine de Rio de Janeiro, au Brésil. Des échantillons de sang ont été prélevés individuellement sur 28 chats d’un programme de castration. L’ADN a été extrait de tous les échantillons et soumis à un séquençage spécifique de *Hepatozoon* spp. L’ADN de *H. felis* a été trouvé chez 21 chats sur 28 (75%) et quatre polymorphismes génétiques, jamais décrits jusqu’à présent, ont été détectés. Il s’agit du premier signalement de *H. felis* chez des chats vivant dans l’État de Rio de Janeiro et les données actuelles confirment que *H. felis* est un complexe d’espèces englobant différents génotypes circulant au sein des populations de chats. Des études supplémentaires sont nécessaires pour déterminer si les différents génotypes ont une biologie ou une pathogénicité différente pour les félidés.

## Introduction

Ticks are vectors of pathogens causing emerging diseases in animals and people worldwide. Most tick-borne diseases (TBDs) are well-known from the epidemiological and clinical standpoints, and this is particularly true for those affecting dogs [21, 42]. Conversely, TBDs of cats are often underestimated and regarded as less important, probably because felines are less susceptible than dogs due to living habits and immunological features [13, 17, 57]. Moreover, many cases of TBDs in cats are often subclinical and the diseases remain undiagnosed and pathogens undetected [11, 12, 42].

However, in recent years, more importance has been attributed to feline TBDs, especially concerning their epidemiology, clinical aspects, and control [7, 14, 47, 49, 52]. Surveys have shown that cats are often positive for pathogens transmitted by ticks in various geographical regions [2, 3, 20, 41, 54, 55] and, among them, *Hepatozoon* spp. have been repeatedly reported in domestic and wild felids in Europe and elsewhere. Cats can be infected by different species of *Hepatozoon*, *i.e. Hepatozoon felis, Hepatozoon silvestris*, and *Hepatozoon canis* [25, 26, 29, 41], and some species could encompass different genotypes. Several genotypes have been identified in domestic and wild cats worldwide [29, 46], and genotypes of *H. felis* may have varying tropism for different felines, as suggested for Bengal tigers, Asiatic lions, Indian leopards, and domestic cats in India [46, 47]. Also, it has been hypothesized that some rare genotypes may occur in small/confined areas and that they may have different degrees of pathogenicity [27, 41].

With regard to South America, findings from the last two decades suggest the existence of a high diversity of *Hepatozoon* genotypes affecting felids [1, 9, 15]. In Brazil, the first records of hepatozoonosis in cats date back almost two decades, when the infection was diagnosed in São Paulo State [49, 53]. The molecular characterization of those isolates has suggested that they are closely related to *H. canis* [53]. Further studies have then shown that domestic and wild felids from other areas of Brazil may be infected by various species or genotypes of *Hepatozoon*, including *H. canis*(*-*like), *H. felis*(*-*like), or isolates close to other species [1, 18, 37, 40]. At present, few data are available on the occurrence of *Hepatozoon* in the State of Rio de Janeiro and they are limited to dogs [23, 43]. No data are available on feline hepatozoonosis in this region of South America, nor on the possible circulation of different species or genotypes of *Hepatozoon* affecting cats. Additionally, the role of individual single *Hepatozoon* species/genotypes in causing various diseases in cats remains to be understood. This study aimed to improve the knowledge of this disease in South America by describing the occurrence and genetic variability of *Hepatozoon* isolates in a population of stray cats.

## Materials and methods

### Animals, sampling, and ethics

Overall, 28 blood samples were collected from cats included in the sterilization program at the Centro de Castração Prefeitura de Niterói (Rio de Janeiro) **–** ethics approval No.: CIAEP 0101482014. All samples were stored in ethylenediaminetetraacetic acid (EDTA) tubes and shipped to the Laboratory of Parasitology of the Department of Veterinary Medicine, University of Teramo, Italy, to be molecularly examined for *Hepatozoon* spp. and *Cytauxzoon* spp. with the permission of Servicio Público Federal of the Universidade Federal Fluminense (LADDP/FV/UFF N°001/2023) and of the Italian Ministry of Health (0004371-15/02/2023-DGSAF-MDS-P).

### Molecular analysis

DNA was extracted from each sample using a commercial kit (Exgene Blood extraction kit, GeneAll Biotech, Songpa-gu Seoul, South Korea), following the manufacturer’s instructions. Specific internal fragments of ~373 bp and of 408 bp of the 18S rRNA gene of *Hepatozoon* spp. and *Cytauxzoon* spp. [44], respectively were PCR-amplified as previously described [41, 44, 60], using appropriate positive controls. All amplicons generated (n. 21, see Results) were purified using a QIAquick^®^ Gel Extraction Kit (QIAGEN, GmbH, Hilden, Germany) and sequenced bidirectionally by a commercial laboratory (Macrogen Italy, Milan, Italy). Sequences were determined in both strands, electropherograms were visually checked to rule out the presence of heteroplasmy [50] or of wrong base calls, aligned, and then compared with each other and with those available in GenBank using the Basic Local Alignment Search Tool (BLAST; http://www.ncbi.nlm.nih.gov/BLAST).

An alignment was produced using MEGAX software [32] and along with the obtained sequences one sequence for each of the 30 *H. felis* haplotypes described by Panda *et al.* (2024) (Accession numbers: ON533605.1; ON075470.1; ON054034.1; OK036954.1; KY056823.1; KX017290.1; KC138533.1; AB636287.1; AB636286.1; AB636285.1; MZ895464.1; OM422756.1; OM462842.1; OM462703.1; OK036961.1; OK036951.1; MZ476769.1; MZ151524.1; MK621310.1; KY511259.1; KU232308.1; AB771577.1; AB771576.1; AB771575.1; AB771574.1; AB7771573.1; AB771572.1; HQ829446.1; HQ829444.1; AY628681.1), 5 sequences of *Hepatozoon americanum* (Accession numbers: EU249992.1; EU249993.1; JX415176.1; AF176836.1; KU729739.1), 1 sequence of *Hepatozoon apri* (Accession number: LC314791.1); 5 sequences of *H. canis* (Accession numbers: KU535870.1; KF322141.1; KF322142.1; KF322143.1; KF322145.1), 2 sequences of *Hepatozoon martis* (Accession numbers: MG136687.1; MG136688.1), 5 sequences of *H. silvestris* (Accession numbers: MH078194.1; KX757031.1; KX757032.1; MF614155.1; KY649445.1); 3 sequences of *Hepatozoon ursi* (Accession numbers: EU041718.1; HQ829429.1; LC431853.1, and finally one sequence of *Karyolysus paradoxa* (Accession number: KX011040.1) and *Haemogregarina podocnemis* (Accession numbers: MF476205.1). The alignment was analyzed in JModelTest [51] to determine the best fitting substitution model to perform a Maximum Likelihood phylogenetic analysis. The evolutionary history was inferred by using the Hasegawa-Kishino-Yano model [28] with discrete Gamma distribution to model evolutionary rate differences among sites. The phylogenetic tree was rooted using *K. paradoxa* and *H. podocnemis* as outgroups.

## Results

DNA of *Hepatozoon felis* was found in 21/28 cats (75%), while none of the samples tested positive for *Cytauxzoon* spp. The sequences were generated from 19 of the 21 *Hepatozoon* amplicons and revealed the presence of five different genotypes. In particular, 11 isolates (1 BH, GenBank Accession Number PP497034) of this study had 100% identity with *H. felis* found in Italy in domestic cats (GenBank Accession Number KY649442.1) [25], Spain (GenBank Accession Number AY628681.1) [15], Israel (GenBank Accession Number KC138534) [5], and Uruguay (GenBank Accession Number MT210598 – Bazzano *et al.*, – unpublished). This genotype had 95.80%–97.55% identity with *H. felis* isolates found in wild felids in Brazil (GenBank Accession Numbers MZ490540, KU232302 and KU232308) [24, 58].

Other sequences herein obtained showed different degrees of identity with the above *H. felis* isolates from Italy, Spain, Israel, and Uruguay, *i.e.* 99.74% (*n* = 2 sequences) (12 BH, GenBank Accession Number PP497035), 99.48% (*n* = 4 sequences) (14 BH, GenBank Accession Number PP497036), 99.22% (*n* = 1 sequence) (20 BH, GenBank Accession Number PP497038) and 98.19% (*n* = 1 sequence) (16 BH, GenBank Accession Number PP497037). Sequence 12 BH, 14 BH, 20 BH, and 16 BH displayed ~95–97% identity with other *H. felis* isolates from Brazil found in an ocelot (GenBank Accession Number MZ490540) [58], and in jaguars (GenBank Accession Numbers KU232308 and KU232302) [24].

**Figure 1:**
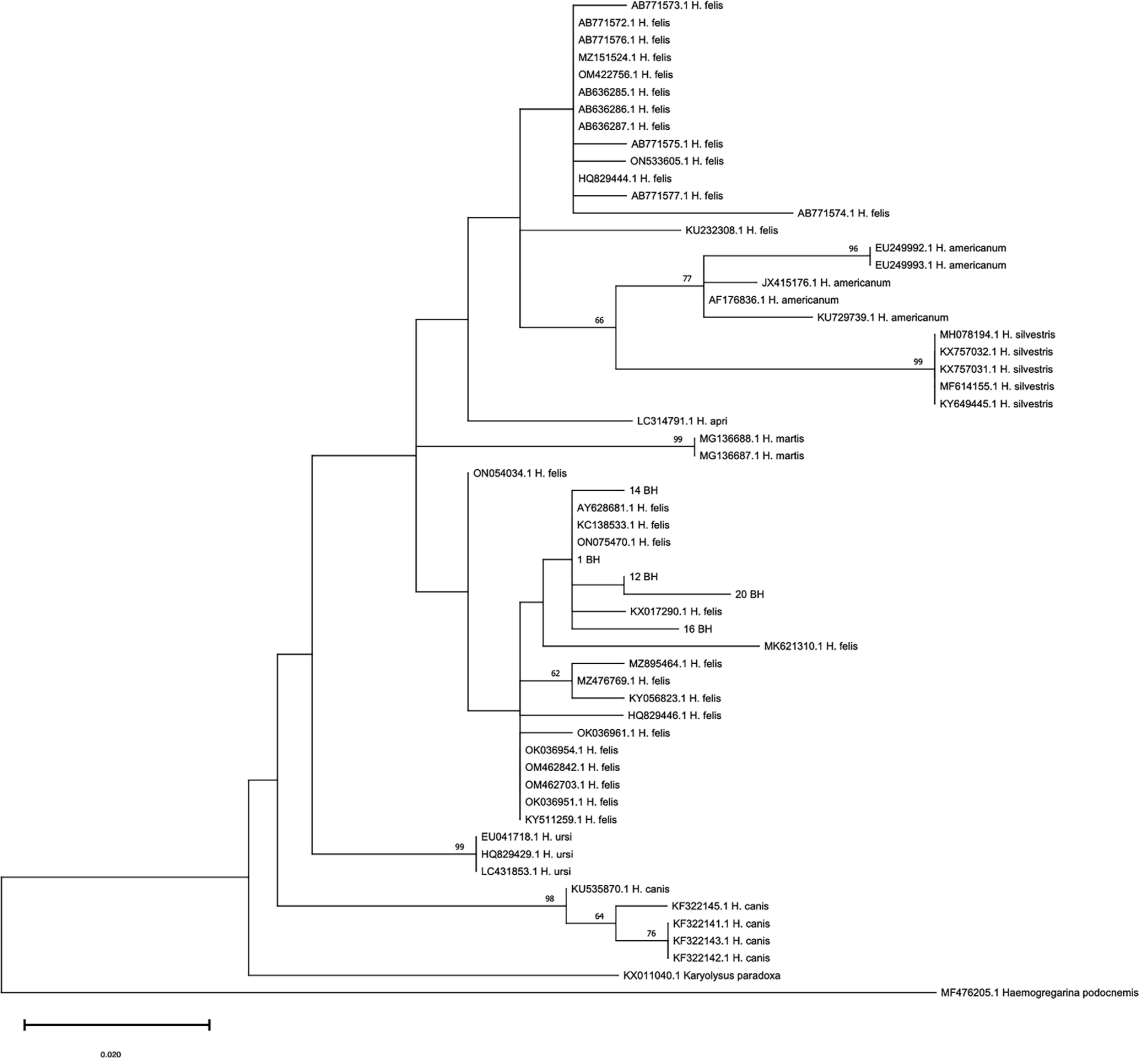
Phylogenetic tree showing relationships between isolates obtained in the present study and sequences used in the recent evolutionary analysis on *Hepatozoon felis* (Panda *et al.*, 2024 [46]). The evolutionary history was inferred by using the Maximum Likelihood method and Hasegawa-Kishino-Yano model. The tree with the highest log likelihood (−783.89) is shown. The percentage of trees in which the associated taxa clustered together is shown above the branches. The tree is drawn to scale, with branch lengths measured in the number of substitutions per site. This analysis involved 58 nucleotide sequences, and there was a total of 184 positions in the final dataset.

The phylogenetic tree showed that all sequences obtained in the present study grouped within the *H. felis* clade 1 described by Panda *et al.*, 2024 [46] (Figure 1). Four new and different genetic polymorphisms were found in sequences of the present study, *i.e.*, 12 BH (*n* = 4 sequences – 1 representative sequence deposited, GenBank Accession Number PP497035), 14 BH (1 sequence, GenBank Accession Number PP497036), 20 BH (1 sequence, GenBank Accession Number PP497038), and 16 BH (1 sequence, GenBank Accession Number PP497037).

## Discussion

To the best of the authors’ knowledge, this is the first description of the genetic variability of *Hepatozoon* spp. affecting domestic cats living in the State of Rio de Janeiro, Brazil.

Epidemiological features of feline hepatozoonosis, including identity of vectors and routes of transmission, have not yet been elucidated.

In analogy to other *Hepatozoon* species, *H. felis* is likely transmitted only by ticks, while alternative modes of transmission (*i.e.*, vertical infections) should also be considered [5, 41]. Studies have shown that hepatozoonosis may occur in different environments, *e.g.*, from highly urbanized settings [20], to dry, wetland, or forested areas [2, 41], as in Brazil [9]. This variability is not surprising considering that *Rhipicephalus sanguineus sensu lato* (*s.l.*), *i.e.*, the most distributed tick in the world for its capability to live and reproduce in several environments [16, 19, 33, 61], is probably one of the main vectors of *H. felis* [8, 54]. Accordingly, *R. sanguineus s.l.* is widespread in Rio de Janeiro State, where it extensively lives in domestic environments [56]. Other ticks that have been suspected to primarily occur in the biology of *Hepatozoon* in cats of Europe are *Ixodes ricinus* and *Rhipicephalus turanicus* [11, 30, 36]. To the best of our knowledge, the presence of *I. ricinus* and *R. turanicus* has never been documented in Brazil (https://datadryad.org/stash/dataset/doi:10.5061/dryad.860473k), but other tick species may be involved in the lifecycle of *H. felis* in this geographic area. *Rhipicephalus sanguineus s.l.* is the tick species that most commonly parasitizes cats in Brazil, including in the State of Rio de Janeiro [10, 22, 38, 39]. Infestations of Brazilian cats by other tick species, *e.g.*, *Amblyomma sculptum* and *Amblyomma aureolatum* [39, 45], have been documented. To date, information on the occurrence of *H. felis* within different tick species in Brazil is lacking, and studies are needed to investigate whether different species or genotypes of *Hepatozoon* infecting felids in Brazil, as elsewhere, are associated with *R. sanguineus* lineages and/or other species of ticks, and what could be the practical implications for their biology, epidemiology and clinical diseases.

The number of samples that were examined molecularly in this study was selected for convenience; therefore, any sound and detailed epidemiological considerations are not possible. Data on infection rates by *Hepatozoon* in populations of domestic cats in Brazil and in South America are scant and a comparison of the present data in terms of percentage of positivity would be difficult. In any case, it is interesting to note that many cat samples examined in a short timeframe were found to be positive for *Hepatozoon*, as previously recorded in other surveys [41]. The grooming behavior of cats as they mechanically remove ticks from their body [17, 34] could lead to vector ingestion, thus favoring the transmission of *Hepatozoon* spp. Moreover, the frequent occurrence of *Hepatozoon* in populations of felids may also be explained by predation, as some species may be transmitted to vertebrates *via* ingestion of prey or ticks feeding on prey [4]. Although this has not yet been proven for *H. felis* or other species/genotypes affecting felids, there is a significant association between hepatozoonosis and outdoor lifestyle in cats, *i.e.*, predation is a likely predisposing factor [5, 6, 35]. Accordingly, all cats infected by *Hepatozoon* in this study were stray animals living in Niterói municipality.

The taxonomical status of *Hepatozoon* spp*.* infecting felids is yet to be clarified. It is proposed that *H. felis* is a species-complex based on a high genetic variability recorded in different studies [27]. Recent findings from Europe, *i.e.*, a new genotype described on a small island of Greece [41], and other phylogenetic analyses have supported this hypothesis [29]. Data from the genetic characterization of *H. felis* genotypes suggest that some are more widespread than others. For instance, one of the most distributed genotypes in various countries of Europe has also been recorded in the Middle East and South Africa [5, 15, 25, 27, 41]. It is thus interesting to note that the latter genotype showed 100% identity with 1 BH found herein, indicating that this genotype is present in South America and present worldwide. Different *Hepatozoon* spp. or more than one genotype of *H. felis* may circulate in cat populations from the same country [27, 29, 41, 46]. The present results are in accordance with this finding, as they showed four different genic polymorphisms never described thus far at the level of the DNA fragment examined, in the small sample of cats studied. Even though the polymorphisms detected here strongly indicate the existence of four new *H. felis* genotypes, this warrants further genetic investigations. Molecular data available in the literature on *Hepatozoon* in felids of South America are scant and derive from studies on wild animals, in which different genotypes have recently been found in jaguars and an ocelot [21, 58]. Accordingly, the present analysis, in particular the phylogenetic relationships of the 1 BH isolate and the sequence KU232308 from wild felids (Figure 1), confirms that different genotypes circulate in felids in Brazil, and suggests the occurrence of an *H. felis* species-complex within the cat population studied here and in felids in South America. On the whole, this scenario from Europe and South America supports the complex-species taxonomic classification recently proposed, and the existence of undescribed separate species [27, 41].

Knowledge on areas endemic for feline hepatozoonosis is of epidemiological relevance for different reasons. Pets travelling with their owners may bring pathogens into free areas or may become infected when visiting endemic regions and bring new pathogens when they return to their region of origin. A constant epidemiological update in these settings is of crucial importance for minimizing the risk that animals may acquire pathogens *via* arthropods, including hepatozoonosis using appropriate prevention measures [6, 31].

Unfortunately, a detailed clinical history was not available for the cats included in the present study, as they were stray cats in a spaying/castration program. However, a good general health condition was assessed prior to the spaying/castration for all the cats (data not shown). In most cases, hepatozoonosis of cats due to *H. felis* is subclinical or a mild disease [5], though cases of severe or fatal signs are described [6]. Considering the variability in *Hepatozoon* infecting felids in various geographical regions, it is plausible that different *Hepatozoon* spp. or *H. felis* genotypes may have different tropism and pathogenicity. For instance, cats may also be infected by *H. silvestris*, a different species with a different tropism within the host (*e.g.*, striated muscle) and a different pathogenic potential, that seem to be higher than *H. felis* [31, 59]. To date, there are no reports of *H. silvestris* in South America.

As discussed elsewhere [41], 1 BH found herein has a ~98% identity with a genotype involved in a severe case of hepatozoonosis in a domestic cat in Austria [6]. Nevertheless, clinical implications based on isolates retrieved in this study cannot be discussed here, as a thorough clinical examination with complete blood analyses was not performed for cats in our study.

In conclusion, this study has provided novel information on the occurrence of *Hepatozoon* spp. infection in Brazil and new comprehensive data on the existence of different genotypes circulating in felids of South America and Europe. Further investigations are needed to ultimately clarify their taxonomical status and whether there is any difference in their relevance and importance in feline clinical practice in terms of epidemiology and pathogenesis.
